# Differential effects of 3,5-T2 and T3 on the gill regeneration and metamorphosis of the *Ambystoma mexicanum* (axolotl)

**DOI:** 10.3389/fendo.2023.1208182

**Published:** 2023-07-10

**Authors:** I. Lazcano, A. Olvera, S. M. Pech-Pool, L. Sachs, N. Buisine, A. Orozco

**Affiliations:** ^1^ Instituto de Neurobiología, Universidad Nacional Autónoma de México (UNAM), Querétaro, Mexico; ^2^ UMR PhyMA CNRS, Muséum National d’Histoire Naturelle, Paris, France; ^3^ Escuela Nacional de Estudios Superiores, Unidad Juriquilla, Universidad Nacional Autónoma de México (UNAM), Querétaro, Mexico

**Keywords:** *Ambystoma mexicanum*, thyroid hormones, metamorphosis, transcriptomics, 3,5-T2, gills

## Abstract

Thyroid hormones (THs) regulate tissue remodeling processes during early- and post-embryonic stages in vertebrates. The Mexican axolotl (*Ambystoma mexicanum*) is a neotenic species that has lost the ability to undergo metamorphosis; however, it can be artificially induced by exogenous administration of thyroxine (T4) and 3,3′,5-triiodo-L-thyronine (T3). Another TH derivative with demonstrative biological effects in fish and mammals is 3,5-diiodo-L-thyronine (3,5-T2). Because the effects of this bioactive TH remains unexplored in other vertebrates, we hypothesized that it could be biologically active in amphibians and, therefore, could induce metamorphosis in axolotl. We performed a 3,5-T2 treatment by immersion and observed that the secondary gills were retracted, similar to the onset stage phenotype; however, tissue regeneration was observed after treatment withdrawal. In contrast, T4 and T3 immersion equimolar treatments as well as a four-fold increase in 3,5-T2 concentration triggered complete metamorphosis. To identify the possible molecular mechanisms that could explain the contrasting reversible or irreversible effects of 3,5-T2 and T3 upon gill retraction, we performed a transcriptomic analysis of differential expression genes in the gills of control, 3,5-T2–treated, and T3-treated axolotls. We found that both THs modify gene expression patterns. T3 regulates 10 times more genes than 3,5-T2, suggesting that the latter has a lower affinity for TH receptors (TRs) or that these hormones could act through different TR isoforms. However, both TH treatments regulated different gene sets known to participate in tissue development and cell cycle processes. In conclusion, 3,5-T2 is a bioactive iodothyronine that promoted partial gill retraction but induced full metamorphosis in higher concentrations. Differential effects on gill retraction after 3,5,-T2 or T3 treatment could be explained by the activation of different clusters of genes related with apoptosis, regeneration, and proliferation; in addition, these effects could be initially mediated by TRs that are expressed in gills. This study showed, for the first time, the 3,5,-T2 bioactivity in a neotenic amphibian.

## Introduction

Metamorphosis is a fascinating phenomenon that occurs in some vertebrate and invertebrate animal species that involves spectacular post-embryonic transition and changes in the morphology, physiology, behavior, and ecology of the individual. Available data suggest that this post-embryonic remodeling is governed by thyroid hormones (THs), at least in chordates (1). A well-studied example is amphibian metamorphosis, in which tadpoles experience a peak of TH plasma levels prior to its onset that triggers tissue remodeling by hierarchically controlling the expression of a complex cascade of target genes (1, 2). The diversity of molecular and cellular processes that convey this life transition involves cell-specific expression and regulation of TH nuclear receptor (TR) genes, THRA and THRB, which act as TH-dependent transcription factors (3–5).

In contrast to frogs, some salamanders like the Mexican axolotl (*Ambystoma mexicanum*) have lost the ability to undergo metamorphosis under natural conditions (6), retaining juvenile characteristics in the mature breeding stage (paedomorphosis) (7). In this neotenic species, plasma TH concentrations are much lower than those found in other premetamorphic anurans, but their tissues possess functional deiodinases and TRs, the molecular components that are essential to decode the TH signal (7). In fact, exogenous exposure to the prohormone T4 or the bioactive T3 experimentally triggers axolotl metamorphosis, confirming the TH involvement in amphibian metamorphosis. However, a more precise characterization of these TH actions is *unclear* given that the protocols used, thus far, have been inconsistent, differing in the method and frequency of hormone administration (immersion or *intra peritoneal (i.p.)* injection), in the developmental stage of the axolotl at the time of induction, among others (8–10).

Recently, 3,5-diiodo-L-thyronine (3,5-T2), an active metabolite of T3, has been shown to act as an endogenous alternative TR ligand with demonstrative biological actions that mimic those of the recognized bioactive T3. For example, 3,5-T2 decreases body weight and serum thyroid- stimulating hormone and regulates lipid metabolism in mammals (11, 12). In fish, both, T3 and 3,5-T2 promote growth (13), and each hormone regulates the expression of specific genes in the tilapia brain and liver (14). Possible thyromimetic effects of 3,5-T2 in other vertebrate species are still unknown. Thus, the aim of the present study was to gain a deeper understanding of TH actions upon axolotl metamorphosis induction, as well as prompted the hypothesis that, as an alternative TR ligand, 3,5-T2 could also induce metamorphosis in this species and/or differentially regulate some aspects of the tissue remodeling involved in this life transition.

## Material and methods

### Animals

Juvenile axolotls were kindly donated by Marco Terrones (Axolkali) and Dr. Jesus Chimal (Instituto de Investigaciones Biomédicas, UNAM). All axolotls were maintained and handled in accordance with protocols approved by the Ethics for Research Committee of the Instituto de Neurobiología, UNAM; the guidelines for use of live amphibians and reptiles in field and laboratory research of the American Society of Ichthyologists and Herpetologists; and the ARRIVE guidelines. Animals of around 8 –10 months after hatching and weighing between 9 and 12 g were maintained at our local housing for at least 20 days prior to experiments at 18°C in a 14-h/10-h light/dark cycle and fed with small pieces of meat and live brine shrimp.

### Metamorphosis induction with thyroid hormone treatment

The immersion protocol allows adding hormones at a desired concentration in the rearing water of aquatic species, eliminating the manipulation stress factor. In the present study, nanomolar concentrations of THs were added to the rearing water and replaced every 3 days. With this TH administration protocol, the onset of metamorphosis at around day 26 post-treatment with T4 was reported (8, 10). Initial experiments to analyze whether 3,5-T2 could induce metamorphosis consisted in treating groups of axolotls (female or male axolotls) with 50 nM of this TH derivative. Control animals were immersed in vehicle (0.001 M NaOH). In further experiments, axolotls were treated with 500 nM of thyroxine (T4), 3,3′,5-L-triiodothyronine (T3) and 3,3′,5′-triiodothyronine (rT3) (Sigma-Aldrich). These treatments were extended for 12 days, a time point at which the rearing water was changed every 3 days for at least 45 days post- withdrawal (dpw). In a second protocol, we immersed axolotls with a higher dose (2 µM) of 3,5-T2 or rT3, and these solutions were replaced with hormone-containing rearing water on Mondays, Wednesdays, and Fridays. The onset of metamorphosis was determined when reabsorption of the external gills and dorsal fin was observed. Climax was established when gills and dorsal fin were completely absorbed. Full metamorphosis was reached when the axolotls became salamanders. Once reaching this state, we observed a 30% increase in lethality, which was unrelated to the hormone used for metamorphosis induction. Phenotype changes in T3-treated axolotls can be observed in **Supplementary Video 1**.

### Semi-quantification of secondary gills length

Secondary gills were measured during the experiments. To this end, we captured microphotographs (dorsal view) of the secondary gills in free moving axolotls by using a stereomicroscope (SteREO Discovery.V12, Zeiss). Subsequently, we measured (software ZEN) at least 10 secondary gill filaments and averaged the length to get a value for each animal. Cases when the first and secondary gills were completely absorbed were reported as “not detected” (n.d.).

### Immunohistochemistry of thyroid hormone receptors in axolotl gill

The complete gill was dissected from a young axolotl previously anesthetized with 0.4% tricaine during 12–14 min. Immediately, the gill was transferred into 4% paraformaldehyde for 24 h, and, then, the tissue was cryoprotected in sucrose (30%) for 12 h and freeze mounted onto aluminum sectioning blocks using Tissue-Tek^®^ O.C.T (Sakura Finetek, Torrance, CA, USA). Sections of ∼ 14 µm were obtained using a cryostat (Leica CM3050, Buffalo Grove, IL, USA) and mounted on Superfrost ™ microscope slides from Fisher Scientific. Gill sections were hydrated in Phosphate Buffered Saline (PBS) during 15 min and then permeated with 2 M HCl during 20 min, and, then, the slides were washed in Tris Buffered Saline (TBS) (3 × 10 min) and treated with citrate buffer 100 mM, pH 6, at 80°C for 30 min, after which free binding sites were blocked with 5% non-fat dry milk (Bio-Rad, Hercules, CA, USA) for 2 h. After blocking, the slides were washed with Tween-Tris Buffered Saline (TTBS) (0.1% Triton X-100 in TBS) three times, and the tissues were incubated for 24 h with the primary anti-thyroid hormone receptor (Abcam, ab42565) in a 1:500 dilution. All the slides were washed (3 × 10 min) and incubated with goat anti-rabbit Immunoglobulin G (IgG) H & L (Alexa Fluor^®^ 488) from Abcam (ab150077) in a 1:1,000 dilution during 12 h. DAPI (4′,6-diamidino-2-phenylindole) was used to label cell nuclei. All the slides were mounted with vectashield (Vector Laboratories Inc., Burlingame, CA, USA), and micrographs were captured with a Zeiss LSM 780 DUO confocal microscope (Carl Zeiss AG, Oberkochen, Germany) and image software ZEN. Image post-processing was performed using ImageJ software (developed by NIH, freeware).

### Differential gene expression analysis

We performed high-throughput sequencing with a differential transcriptomic analysis on the gills of control, 3,5-T2–treated, and T3-treated juvenile axolotls. The animals were submitted to an immersion protocol with vehicle, 500 nM of 3,5-T2 or T3 of by 6 days. At this time point, the gills were dissected in axolotls anesthetized with 0.4% tricaine. Immediately, the gill was transferred into RNA later (Invitrogen), manually homogenized, and processed for total RNA extraction using TRIzol (Invitrogen).

Libraries were generated using the Illumina TruSeq RNA Sample Preparation Kit according to the manufacturer’s instructions. Transcriptome sequencing was conducted using Genome Analyzer GAIIx (Illumina) at the genome sequencing facility of our university located at “Instituto de Biotecnología, UNAM”. A configuration for pair-end reads with a 72-bp read length was used. Sequence Read Archive data are available as BioProject ID: PRJNA957439.

Reads quality control were generated with FastQC (www.bioinformatics.babraham.ac.uk/projects/fastqc/) and mapped on axolotl genome (GCA_002915635.3_AmbMex60DD) with bowtie2 (15) ran with sensitive parameters (–sensitive). Differential analysis and clustering followed the same procedure as previously described (16).

Enrichment analysis of biological processes and pathways are based on the GORILLA website (17) and Kyoto Encyclopedia of Genes and Genomes (KEGG) pathways databases (18). The protein–protein interaction network was modeled from the BIOGRID database (19).

### RT-qPCR verification of transcriptomic data

The mRNA was reversed -transcribed (RT) (RevertAid First Strand, Thermo Fisher) from 0.5 µg of total RNA using an oligo(dT) primer (final volume of 20 µl). Quantitative 2^−ΔΔCT^ qPCR was carried out in duplicates in two independent assays with three samples per group using the geometric mean of Gapdh and Eif5a as an internal standard in reactions that contained 2 µl of the diluted 1:10 RT reaction, 3.3  µl of Maxima SYBR Green/ROX qPCR Master Mix (Fermentas, Waltham, MA, USA), and 250  nM forward and reverse primers in a final volume of 8 µl. PCR protocol of 95°C for 10 s, 60°C for 10 s, and 72°C for 10 s were used. Oligonucleotides used are enlisted in **Supplementary Data 2**.

### Data analysis

Results of the length of secondary gills are presented as the mean ± S.E.M. Data were analyzed using one-way ANOVA followed by a Tukey *post -hoc* test. Values of *p* < 0.05 were considered statistically significant.

## Results

### Effect of the different THs on axolotl metamorphosis

**Figure 1:**
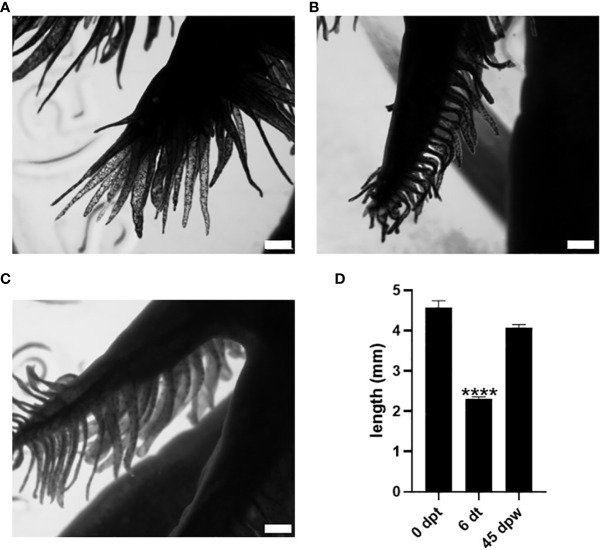
Effect of 50 nM 3,5-T2 exposure upon the length and morphology of axolotl secondary gills. Dorsal view photomicrographs of axolotl secondary gill were taken to analyze the effect of the 3,5-T2 immersion treatments upon the length of the gill at 0 dpt. **(A)** Six days after the onset of 3,5-T2 treatment (6 dt). **(B)** Forty-five days post-withdrawal (dpw) of 3,5-T2 exposure. **(C)** The semi-quantification of secondary gill lengths from **(A)** to **(C)** is shown in **(D)**; data are represented as the mean ± S.E.M. (n = 4) and were analyzed using one-way ANOVA followed by a Tukey *post -hoc* test. Asterisks indicate statistical difference when compared to 0 dpt. **** *p* < 0.01. Scale bar, 1 mm.

**Figure 2:**
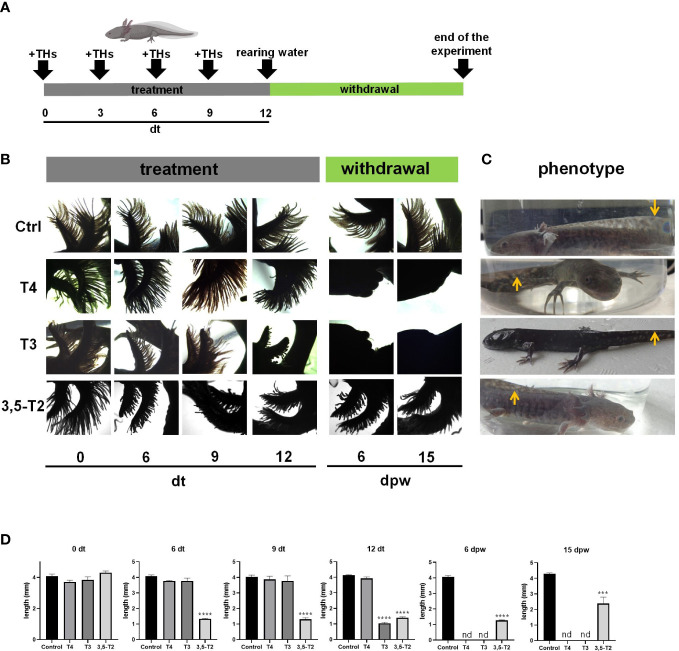
Effects of different iodothyronines on the length and morphology of axolotl secondary gills. **(A)** Graphical representation of the experimental protocol, including TH treatment and withdrawal. **(B)** Dorsal view photomicrographs of axolotl secondary gill treated with vehicle (Ctrl) or 500 nM of T4, T3, or 3,5-T2. Days of treatment (dt) or post- withdrawal (dpw) are indicated. **(C)** Phenotype of the treated axolotl. After 45 dpw, control and 3,5-T2– treated animals showed the classical neotenic phenotype; T4 treatment only reached a climax phenotype, and T3- treated animals were post-metamorphic. Arrows indicate the similarities and differences of the dorsal fin. **(D)** The semi-quantification of secondary gill lengths from **(B)**. Pictures show the morphology of the axolotls under different treatments at 23 dpw. Data are shown as mean ± S.E.M. (n = 3 per group). In **(D)**, data were analyzed using one-way ANOVA followed by a Tukey *post- hoc* test. Asterisks indicate statistical differences between treatment and control groups. ***p < 0.01; **** *p* < 0.001; n.d., not detected.

As an initial approach to test whether 3,5-T2 could induce axolotl metamorphosis, we used an immersion protocol previously characterized for T4 (8, 10), but no induction was observed with the 50 nM 3,5-T2 treatment (data not shown). After a 10-fold increase in 3,5-T2 concentration (500 nM), secondary gill retraction (~ 50%) was observed 6 days after the onset of the treatment (**Figures 1A, B**). As time progressed, no further morphological changes that indicated metamorphosis progression were observed in the treated axolotl, but secondary gill retraction was more evident, prompting withdrawal at day 25 post- treatment to avoid the possible death of the individuals. Unexpectedly, progressive secondary gill regeneration was observed, recovering their full-length around 45 dpw (**Figures 1C, D**). The reversible gill phenotypic transition promoted by the 3,5-T2 treatment withdrawal raised the possibility that these effects were also elicited by the prohormone T4 and the bioactive T3. Therefore, we replicated experiments treating axolotls with equimolar concentrations of 500 nM of T4, T3, or 3,5-T2 for 12 days, when visible changes in gill retraction were evident in the 3,5-T2–treated group. At this time point, we withdrew all hormone treatments and close monitored the axolotl for phenotypic changes for 23 dpw (**Figure 2A**). For this experiment, a more systematic characterization of the TH-specific effects upon axolotl metamorphosis was achieved, identifying pre-metamorphic, onset, climax, and post-metamorphic stages (**Supplementary Figure 1**; **Supplementary Video 1**). As shown in **Figures 2B, D**, only 3,5-T2 treatment elicited secondary gill retraction by 6 days post- treatment (dpt), whereas exposure to T3 elicited this retraction after 12 dpt and T4 effects were only observed after 3 dpw. Furthermore, as previously observed, suspension of 3,5-T2 treatment elicited secondary gill regeneration, whereas an irreversible metamorphic progression resulted from T4 or T3 withdrawal (**Figures 2B, D**). The T3-treated group exhibited a full post-metamorphic (salamander) phenotype by 23 dpw, whereas that of T4 presented a climax stage phenotype at this same time point (**Figure 2C**).

### 3,5-T2 can induce axolotl metamorphosis

**Figure 3:**
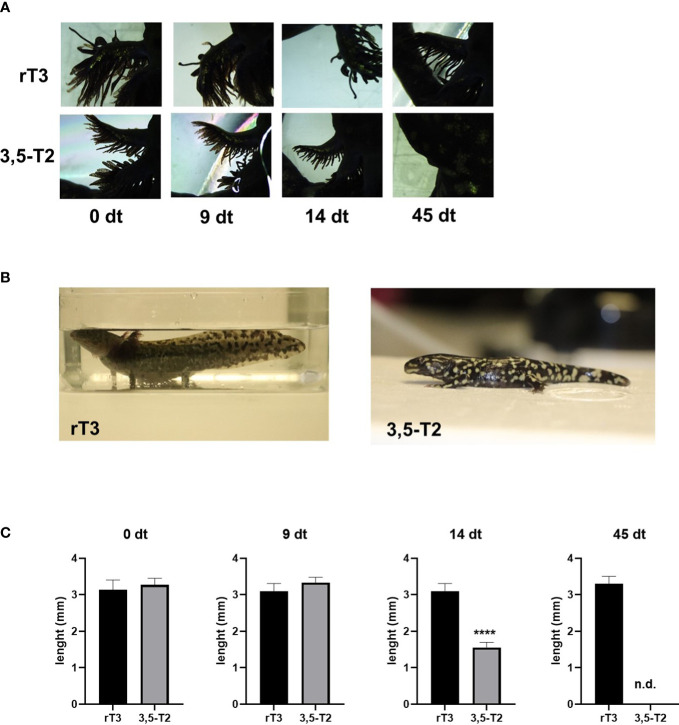
3,5-T2 induces metamorphosis in axolotls. **(A)** Dorsal view photomicrographs of secondary gill from axolotls treated with 2 µM of 3,5-T2 or rT3. Days of treatment (dt) are indicated. **(B)** Axolotl phenotypes at the end of the experiment (45 dpt), showing a 3,5-T2 but not by rT3 metamorphosis induction. **(C)** Semi-quantification of the length of secondary gills from **(A)**; data were analyzed using one-way ANOVA followed by a Tukey *post- hoc* test and are shown as the mean ± S.E.M. (n = 3 per group). Asterisks indicate statistical differences between groups. ****p < 0.001. n.d., not detected.

Given that some of the TH-related effects observed in mammals generally require higher concentrations of 3,5-T2 than those of T3 (11), we tested whether a higher 3,5-T2 concentration (2 µM) could induce axolotl metamorphosis using the same immersion protocol (**Figure 3A**). In addition, we included a negative control group treated with equimolar concentrations of the inactive reverse T3 (rT3) (20). Interestingly, 3,5-T2 was able to induce complete metamorphosis, evidencing that, for the first time, this alternative ligand is bioactive also in the axolotl, whereas rT3 lacked metamorphic effects, further confirming its inactive nature in this species, at least in this experimental paradigm (**Figures 3B, C**). Unexpectedly, and although 3,5-T2 and T3 were able to induce full metamorphosis, the resulting phenotypes of the salamanders were different, particularly regarding the skin color pattern. 3,5-T2– triggered metamorphosis produced salamanders with intense and abundant yellow spots, whereas the skin of the T3-treated group was darker and almost spotless (**Supplementary Figure 2**).

### Axolotl gill expresses thyroid hormone receptors

**Figure 4:**
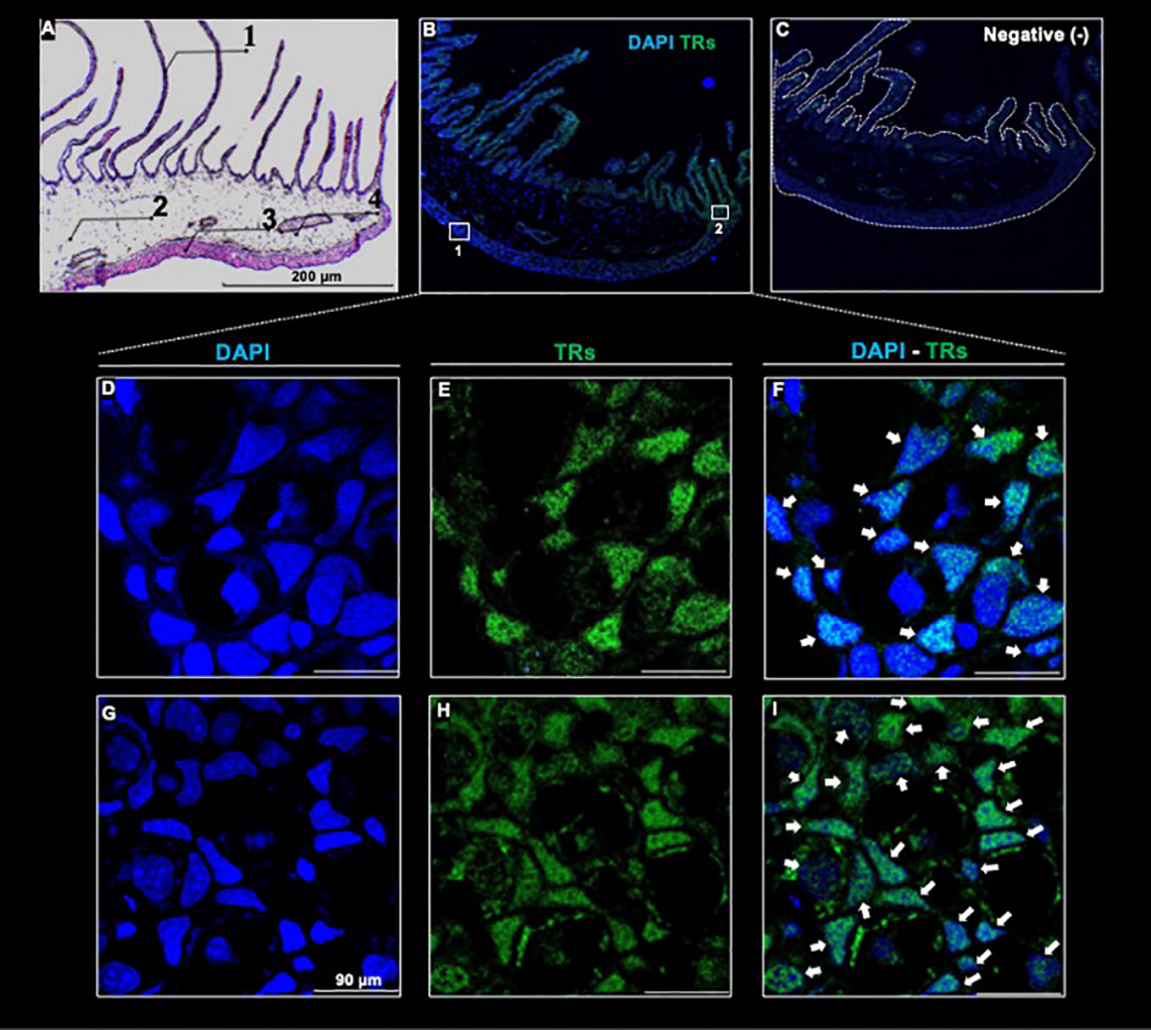
Thyroid hormone receptor immunodetection in a full-length transversal axolotl gill **(A)** Hematoxylin/eosin staining. Numbers indicate gill filament (1); gill mesenchyme (2); external portion of the gill (3), and gill muscle (4). **(B)** Co-localization of anti - TR/DAPI. **(C)** Negative control. **(D–F)**: amplification of the external gill, **(G–I)**: amplification of the filament base. White arrows indicate anti - TR immunoreactive cells. Scale bar = 90 µm

The observed reversible gill phenotypic transition elicited by 3,5-T2 treatment withdrawal raised the question of whether 3,5-T2 effects were indeed mediated by canonical TRs. To investigate this, we first explored secondary gill cyto-architecture and observed the presence of a large number of filaments along the tissue, a gill mesenchyme, followed by a layer of undetermined cells and of gill muscle (**Figure 4A**). In addition, we employed a specific antibody, which is predicted to bind to axolotl TRα and TRβ isoforms (**Supplementary Figure 3**). The immunohistochemistry revealed the presence of TRs mainly in the secondary gills and in the tissue opposite to these filaments (**Figures 4B, C**). While exploring in more detail the subcellular TR distribution, we detected a strong immunoreactivity signal co-localizing with the nuclei of these receptors in both structure samples [**Figures 4D–I** (1), I (2) ], suggesting that 3,5-T2 could be signaling through canonical TRs.

### 3,5-T2 and T3 regulate different gene networks in the gill

**Figure 5:**
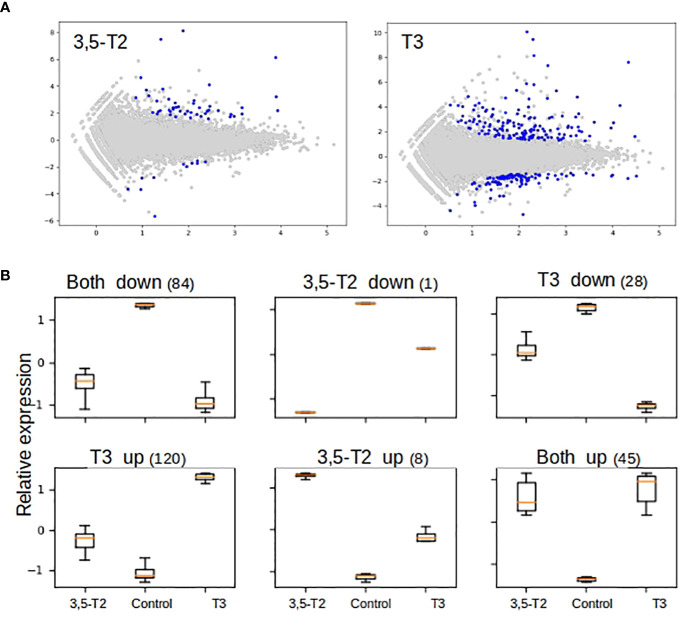
Differential gene expression and cluster analysis in the axolotl gill after 3,5-T2 or T3 treatment. **(A)** MA plot of differentially regulated genes by 3,5-T2 or T3. Data represent individual gene responses plotted as logarithmic fold-change vs. total counts of mRNA, False Discovery Rate (FDR) < 0.05. **(B)** Cluster analysis of logarithmic fold change of gene expression normalized and scaled (*y*-axis), comparing TH treatments vs. controls (*x*-axis). Number above represents the total number of genes in each cluster.

With the aim of analyzing the possible molecular pathways involved in secondary gill remodeling after 3,5-T2 or T3 exposure, we treated axolotl with 500 nM of either hormone for 6 days, and gill total RNA was extracted to perform a high-throughput transcriptome sequencing followed by differential analysis. An illustrative example of quality control as well as sequencing and mapping statistics are shown in the **Supplementary Data 1**. Independent RT-qPCR measures of gene expression changes on a selected number of target genes were in very good agreement with RNA- seq data (**Supplementary Data 2**), thus illustrating the robustness of our analyses. We next conducted a cluster analysis on normalized expression levels (**Figure 5A**) to derive groups of synexpression and compared T3- or 3,5-T2–treated axolotl versus controls. As discussed previously (16, 21), the direct comparison of differential analysis results (e.g., with Venn diagrams) often fails because it relies too much on p-values set in an unfavorable statistical setting (low number of biological replicates and high number of observables). On the basis of this analysis, we found a total of 138 and 277 genes regulated by 3,5-T2 and T3, respectively. Note that nine genes are regulated by 3,5-T2 alone and 148 genes by T3, thereby readily identifying specific components of gene regulatory responses (**Figure 5B**). The full list of gene clusters is provided in **Supplementary Data 3**. It is interesting that the cluster of T3 exclusively upregulated genes is the largest, comprising 120 genes, almost three times more than those upregulated by both iodothyronines (45 genes).

**Figure 6:**
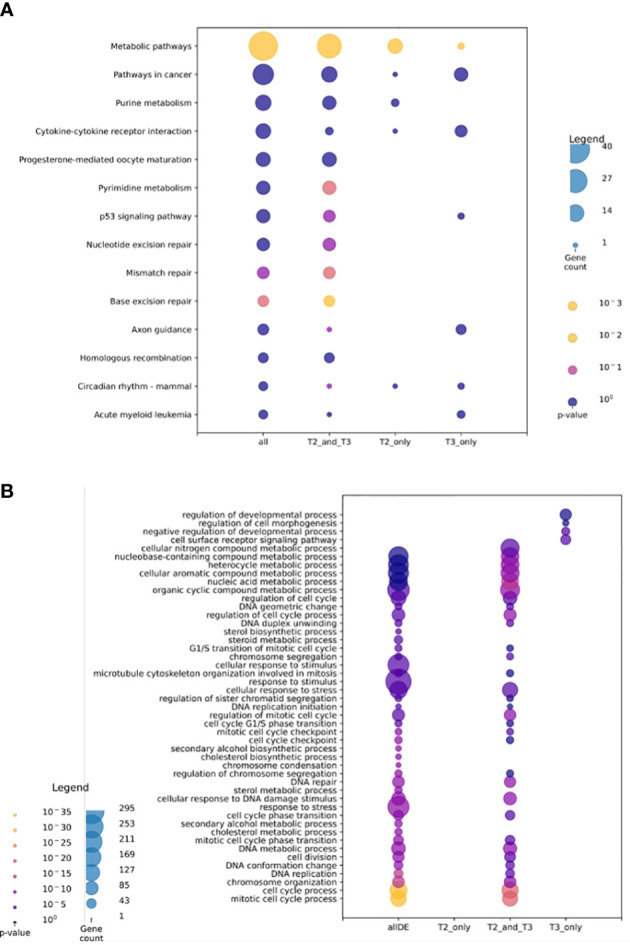
Gene ontology analysis for transcriptomic data. **(A)** Gene ontology UniProt–Gene Ontology most enriched biological processes. **(B)** Analysis of KEGG most enriched pathways. Sets **(A, B)** show all transcriptomic data; 3,5-T2 and T3 regulated transcripts, and 3,5-T2– or T3-exclusively regulated transcripts. The size of the circle represents the transcript abundance as gene count and color represents the *p-* value.

We performed a gene ontology analysis to explore the physiological implications of T3 and 3,5-T2 treatments upon gill remodeling (**Figure 6**). The most enriched biological process found for genes regulated by both hormones belonged to three principal categories: metabolism, cell cycle, and stress. The lists of genes regulated exclusively by 3,5-T2 or T3 did not show significant enrichment of biological processes, as often found when the number of genes is low (22). Nevertheless, some of the genes exclusively regulated by T3 have been associated with developmental processes. The analysis of KEGG revealed that metabolism, cell signaling, and cell cycle are pathways activated by genes regulated by both iodothyronines (**Figure 6B**). Interestingly, the p53 pathway was identified in the pool of genes exclusively regulated by T3; this gene network could be participating either in cell cycle arrest or in apoptosis. This made us think that the differences observed in metamorphosis induction, that is, reversible (+ 3,5-T2) or full secondary gill absorption (+ T3), could be operated by an interplay of tissue remodeling, proliferation, and apoptosis. This idea was further supported by the manual identification of other 3,5-T2– and T3-differentially regulated genes, all of which are known to participate in processes that are key for life transition events, like metamorphosis (**Table 1**).

**Table 1:** Genes implicated in tissue remodeling regulated specifically by 3,5-T2 or T3.

3,5-T2 regulated genes		
FGF23	Fibroblast growth factor 23; this gene encodes a member of the fibroblast growth factor family of proteins, which possess broad mitogenic and cell survival activities. ^(2)^	Upregulated
KY	Kyphoscoliosis peptidase; this protein is involved in the function, maturation, and stabilization of the neuromuscular junction and may be required for normal muscle growth. ^(2)^	Downregulated
T3 regulated genes		
*Gene symbol*	*Function summary*	*Regulation*
NOXO1	NADPH oxidase (NOX) organizer; the *knockout* increases of the proliferative capacity in colon epithelial cells. ^(1)^	Upregulated
MMP11	Matrix metalloproteinase 11; this protein family is involved in the breakdown of extracellular matrix in normal physiological processes, such as embryonic development, reproduction, and tissue remodeling. ^(2)^	
SORBS3	This gene encodes an SH3 domain–containing adaptor protein. The presence of SH3 domains play a role in this protein’s ability to bind other cytoplasmic molecules and contribute to cystoskeletal organization, cell adhesion and migration, signaling, and gene expression. ^(2)^	
ADAMTS17	A disintegrin-like and metalloprotease; this has diverse roles in tissue morphogenesis in xemopus development. ^(3)^	
CDKN1B	This gene encodes a cyclin-dependent kinase inhibitor and controls the cell cycle progression at G1. ^(2)^	
NGFR	Nerve growth factor receptor; this plays an important role in differentiation and survival of specific neuronal populations. ^(2)^	
BCOR	BCL6-interacting corepressor; a POZ/zinc finger transcription repressor is required for germinal center formation and may influence apoptosis. ^(2)^	
APCDD1L	Adenomatosis polyposis coli down-regulated 1 protein-like; this predicted to be involved in negative regulation of Wnt signaling pathway. ^(2)^	
MMP28	Matrix metalloproteinase 23B; this family is involved in the breakdown of extracellular matrix. ^(2)^	Downregulated
FAM107A	Family with sequence similarity 107 member A; this is involved in several processes, including negative regulation of G1/S transition of mitotic cell cycle; negative regulation of focal adhesion assembly; and regulation of cytoskeleton organization. ^(2)^	
PITX1	Paired-like homeodomain transcription factor 1; this transcriptional regulator is involved in organ development. ^(2)^	
CARD10	Caspase recruitment domain–containing protein 10; this participates in apoptosis signaling through highly specific protein-protein homophilic interactions. ^(2)^	
MELK	Maternal embryonic leucine zipper kinase; this is involved in apoptotic process, cell population proliferation, and protein autophosphorylation. ^(2)^	

^(1)^ Moll et al., 2018 (https://doi.org/10.3389/fimmu.2018.00973)

^(2)^ GeneCards.

^(3)^ Desanilis et al., 2018.

**Figure 7:**
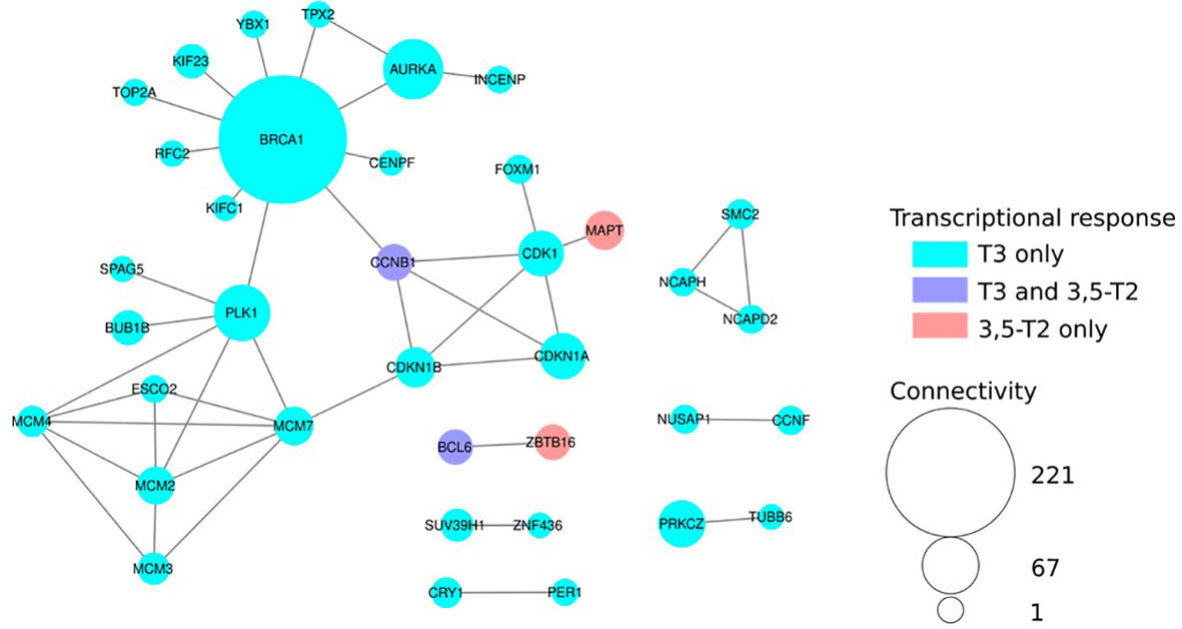
Network analysis of Differentially Expressed (DE) genes reveals hot spots of THs action. Protein–protein interaction network build from the BIOGRID database. Nodes colored in cyan are DE only after T3 treatment and red only with 3,5-T2. Violet nodes are DE with both T3 and 3,5-T2. Node size is proportional to its connectivity, i.e., the number of known protein partners it interacts with. Large nodes (hubs, connectivity > 20) are major sensors and controllers of biological networks. For clarity, only DE genes connected to other DE genes are shown.

We also explored whether 3,5-T2 or T3 differentially regulated genes engaged in particular known protein–protein interactions within biological networks (BioGRID analysis). We found a major component constituted of highly interconnected nodes (i.e., gene products) mostly involved in DNA replication, cell cycle control, and chromatin dynamics, further illustrating TH functional input in these cellular processes (**Figure 7**).

## Discussion

In the present study, we describe that, for the first time, 3,5-T2 can induce axolotl metamorphosis and that this alternative TR ligand elicits transient and reversible gill remodeling that involves the regulation of specific gene clusters.

3,5-T2 has been well recognized as an alternate TR ligand (Mendoza et al., 2013); it regulates TH canonical actions like growth (13, 23) and lipid metabolism (12, 24), as well as elicits differential effects upon the expression of TH-regulated genes in fish (23, 25) and mammals (11, 12, 24). Other TH alternative ligands had been shown to induce metamorphosis in several species that emerged in different timepoints of evolution. For example, Triiodothyroacetic acid (TRIAC) has been shown to promote amphioxus metamorphosis (26, 27). However, whether 3,5-T2 had postembryonic remodeling effects in any amphibian had never been explored. In this context, the paedomorphic axolotl is a fascinating model because, in contrast to anurans, they can undergo metamorphosis only after T4 or T3 exogenous treatment (8–10). When treating axolotl with equimolar concentrations (500 nM) of T4, T3, and 3,5-T2, only T4 and T3 were capable of inducing full metamorphosis. Interestingly, T4 induction occurred around 20 days later than that with T3. This highlights the prohormone nature of T4, which requires a biotransformation into the bioactive form of the hormone, possibly catalyzed by deiodinase type II (28). T4 could act as a partial TR agonist (29), resulting in a full but time extended transformation. In contrast, exogenous administration of T3 directly promotes cellular actions and induces a faster metamorphosis.

Interestingly, immersion in 500 nM 3,5-T2 only affected the size of the secondary gills without inducing a true metamorphosis, whereas treatment withdrawal resulted in progressive secondary gill regeneration. This suggests differential effects of 3,5-T2 at least in this remodeling process. The lack of metamorphosis induction at this concentration could be either a reflection of deficient intracellular TH transport and/or a lower 3,5-T2 affinity for TRs as has been described for mammals (11). Neither of these mechanisms have been studied in amphibians for any TH or their derivatives, leaving these hypotheses still unresolved. Full axolotl metamorphosis was attained when the 3,5-T2 treatment was scaled four-fold. As compared with T3, 3,5-T2 has a higher affinity for L-TRβ1 in fish and lower affinity for TRβ1 in humans, respectively (30), and thus preferentially binds to the beta isoform. Al though we do not know whether 3,5-T2 is binding to canonical axolotl TRs, our results show that the secondary gill expresses these receptors abundantly, which could explain the early effects on gill retraction with all tested iodothyronines. Gaining insight of 3,5-T2 and T3 binding affinity for axolotl TR isoforms would be necessary to analyze these hypotheses.

A T3- or 3,5-T2–treated gill transcriptomic analysis was performed to further decipher if the expression of different gene clusters could explain the observed effects upon secondary gill remodeling processes. Our results showed that both iodothyronines induced a change in gene expression; these findings, together with the notion that TRs are being expressed in the gill, allow the hypothesis that 3,5-T2 and T3 are acting through canonic TR isoforms to modulate tissue remodeling. 3,5-T2-exclusively regulated genes were 10 times lower than those T3-exclusively regulated, suggesting that these hormones could be acting through different TR isoforms that have divergent outcomes. Furthermore, T3 seems to be irreversibly involved in tissue remodeling, regulating genes such as metalloproteinases like the matrix metalloproteinase 11 (MMP11) and the ADAM metallopeptidase with thrombospondin type 1 motif 17 (ADAMT17). MMP11has been shown to be a T3 target in the Xenopus laevis metamorphosis, essential for the degradation of the extracellular matrix during tissue remodeling and ADAMT17 involved on tissue morphogenesis in Xenopus development (31, 32). Another possibility to explain the more pronounced effects elicited by T3 is that this hormone is regulating the expression of other transcription modulators that, in turn, regulate the expression of different gene sets, as the *HR lysine demethylase* and *nuclear receptor corepressor* as well as *the Bcl6 corepressor* (BCOR), found in the T3 upregulated gene cluster.

The fact that 3,5-T2 withdrawal results in gill regeneration is puzzling and suggests that this hormone does not fully engage with the irreversible gill remodeling program triggered by equimolar concentrations of T3. This is certainly enigmatic and, at the moment, difficult to elucidate. Indeed, a smaller number of gene clusters are regulated by this hormone; some of these genes are still not fully annotated, leaving the possibility that they could participate in proliferative or mitogenic processes.

Protein–protein interaction differences within biological networks after 3,5-T2 or T3 treatments also reflect the transcriptomic analysis outcomes. Indeed, TH functional input is mostly involved in DNA replication, cell cycle control, and chromatin dynamics. Interestingly, among the additional small subnetworks, two transcription factors, Bcl6 and Zbtb16, display a differential regulation after T3 or 3,5-T2 treatments, a thought-provoking result, given that Zbtb16 is a repressor of Bcl6 (33, 34), suggesting opposing TH-regulating cell fate mechanisms.

Understanding the molecular mechanisms that underlay the pleiotropic effects exerted by THs is a current challenge in thyroid physiology. The combination of tissue-specific expression TR isoforms and the bioavailability of different TH derivatives are some of the events that have explained, in part, this pleiotropy. This is clearly illustrated by the very specific effects (i.e., reversible gill absorption and different skin phenotype in post-metamorphic axolotl) elicited by 3,5-T2 in a very complex developmental phenomenon. From a translational viewpoint, our observations add further support to the notion that alternative TR ligands could be used to pharmacologically target specific actions in illness.

## Data availability statement

The datasets presented in this study can be found in online repositories. The names of the repository/repositories and accession number(s) can be found in the article/**Supplementary Material**.

## Ethics statement

All axolotls were maintained and handled in accordance with protocols approved by the Ethics for Research Committee of the Instituto de Neurobiología at the Universidad Nacional Autónoma de México (UNAM).

## Author contributions

AOr: Conceptualization, Planning, Writing, Funding, Review and Discussion. IL; Conceptualization, Planning, Writing, Experimentation, Funding, Review and Discussion. AOl: Transcriptomic analysis and validation, Review and Discussion. SP-P: Immunohistochemical analysis, Review and Discussion. LS: Manuscript Review. NB: Transcriptomic analysis, Review and Discussion. All authors contributed to the article and approved the submitted version.
