# Supplementary material for: Differential effects of 3,5-T2 and T3 on the gill regeneration and metamorphosis of the Ambystoma mexicanum (axolotl)
Source: Front Endocrinol (Lausanne). 2023 Jul 10;14:1208182. doi: 10.3389/fendo.2023.1208182 (PMC10364608; doi:10.3389/fendo.2023.1208182)
Supplement: Supplementary file 2 [file DataSheet_1.pdf]

A

Library	Reads	Mapped	%
Ctrl_1	11,308,460	4,020,613	35.6
Ctrl_2	7,620,058	4,040,368	53.0
Ctrl_3	9,773,840	4,073,542	41.7
T2_1	8,405,953	4,174,309	49.7
T2_2	8,298,677	4,207,122	50.7
T2_3	8,147,493	4,241,801	52.1
T3_1	9,469,361	4,576,449	48.3
T3_2	9,413,018	5,010,879	53.2
T3_3	8,489,772	6,635,497	78.2

B

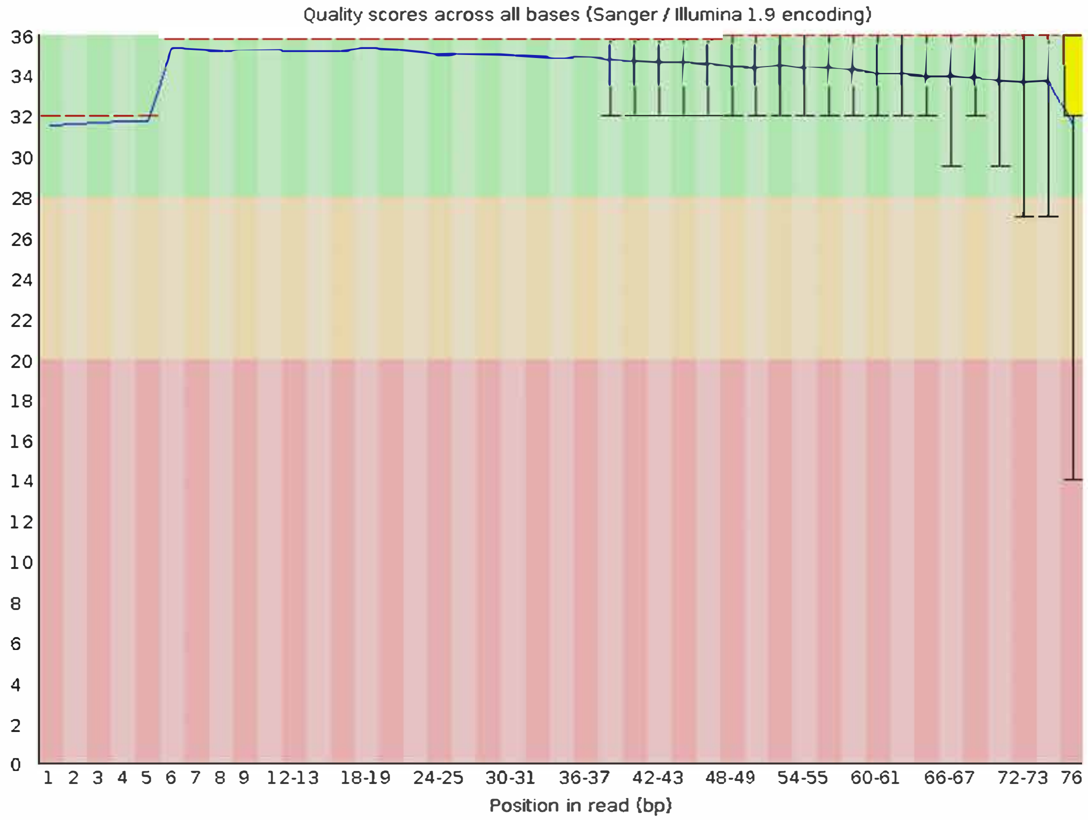

C

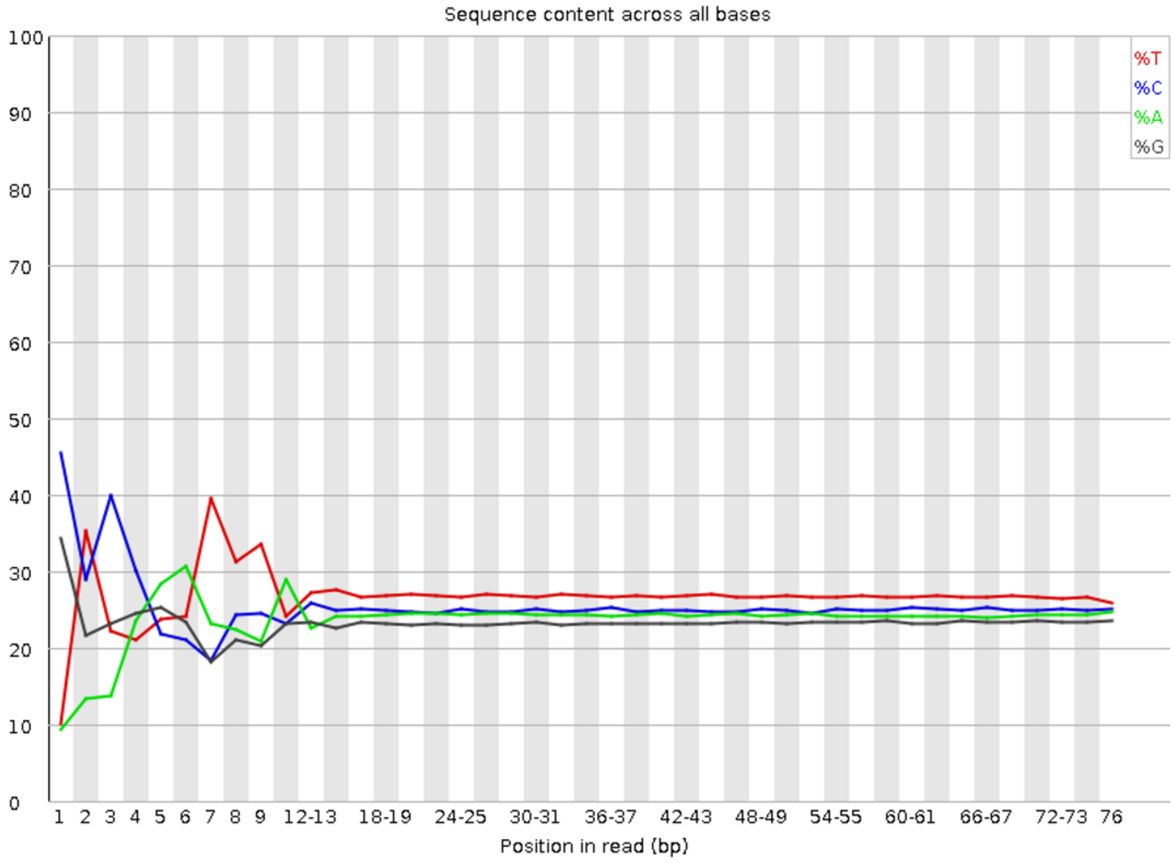

Supplemental Data 1. Raw data quality controls. A. Reads statistics. **B**. Typical example of quality score distribution. For each position, score dispersion among all reads is displayed as a box plot, and the median is shown in red. The highest quality is reached at score = 36. Green, orange and red areas corresponds to score of high, medium and low quality. **C**. Nucleotidic content across all bases. This is expected to be random for each position (flattened lines). The non-random pattern between position 1 to 13 indicates sequencing primers contamination, which had to be clipped before further data processing.
