# Supplementary material for: Differential effects of 3,5-T2 and T3 on the gill regeneration and metamorphosis of the Ambystoma mexicanum (axolotl)
Source: Front Endocrinol (Lausanne). 2023 Jul 10;14:1208182. doi: 10.3389/fendo.2023.1208182 (PMC10364608; doi:10.3389/fendo.2023.1208182)
Supplement: Supplementary file 3 [file DataSheet_2.pdf]

A

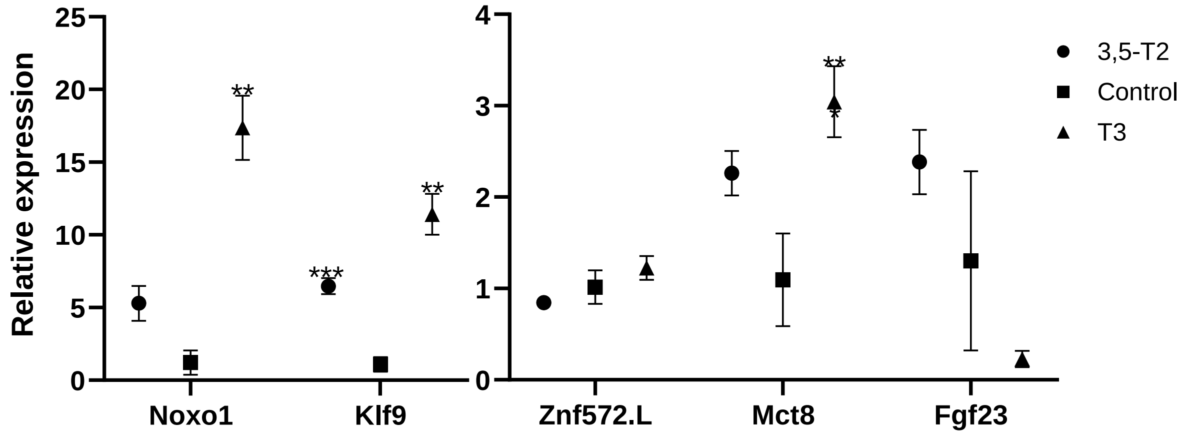

RNA-seq	Noxo1	Klf9	Znf572.L	Mct8	Fgf23
3,5-T2 Log2FC	-0.4	2.86	0.55	0.05	1.28
T3 Log2FC	1.37	2.94	1.21	1.28	-1.14

B

Gene symbol	Gene ID	Primer sequence		Amplicon (bp)	Ta (°C)	R^2
Noxo1	AMEX60DD020810	Fw:	GGAAGGCAGAAGACGTACATG	200	60	0.94
		Rv:	CGGTTGGACCTCCTGAATAGAA			
Klf9	AMEX60DD043157	Fw:	GGTGCACACAGGTGAACGA	188	60	0.98
		Rv:	ATTCAGTGTGACGACGAGCG			
Znf572.L	AMEX60DD032441	Fw:	TCTCAGCAGGAAGCCCTGTT	166	60	0.97
		Rv:	ATCCGCAAGAGGATATCGGG			
Fgf23	AMEX60DD006585	Fw:	CCAAACCATTTACAGTGCGCT	194	60	0.97
		Rv:	TGGGGGAGTGGTAGACATCA			
Mct8	AMEX60DD037055	Fw:	GCTCCTTCACAAGGTCTCTTG	183	60	0.98
		Rv:	AGAAAGGGAGGGTCATCGTG			
Gapdh	Guelke et al., 2015	Fw:	CGTGACCCCGCCAACATC	117	60	0.96
		Rv:	ACGCTTAGCACCGCCCTTCA			
Eif5a		Fw:	GCCGGGGCTTCCACCAC	126	60	0.99
		Rv:	ATGACCATGCTTGCCAGTTTTG			

Supplemental Data 2. RT-qPCR validation of transcriptome selected genes. A) A group of 5 differentially expressed genes (see above) was selected for further validation by RT-qPCR, mRNA relative expression showed as $2^{-\Delta\Delta CT}$ in which we used the geometrical mean of two housekeeping genes as internal standard; and compared with the logarithmic 2 fold change in the RNA-seq data. RT-qPCR values are means of +/- SEM of 2 independent assays and significance denoted as *p*< *0.05, **0.005, ***0.0005 after one-way ANOVA analysis with Bonferrroni *post-hoc* test. **B)** Primers sequence and RT-qPCR specifications for selected genes.
