# Supplementary material for: Differential effects of 3,5-T2 and T3 on the gill regeneration and metamorphosis of the Ambystoma mexicanum (axolotl)
Source: Front Endocrinol (Lausanne). 2023 Jul 10;14:1208182. doi: 10.3389/fendo.2023.1208182 (PMC10364608; doi:10.3389/fendo.2023.1208182)
Supplement: Supplementary file 5 [file Image_1.pdf]

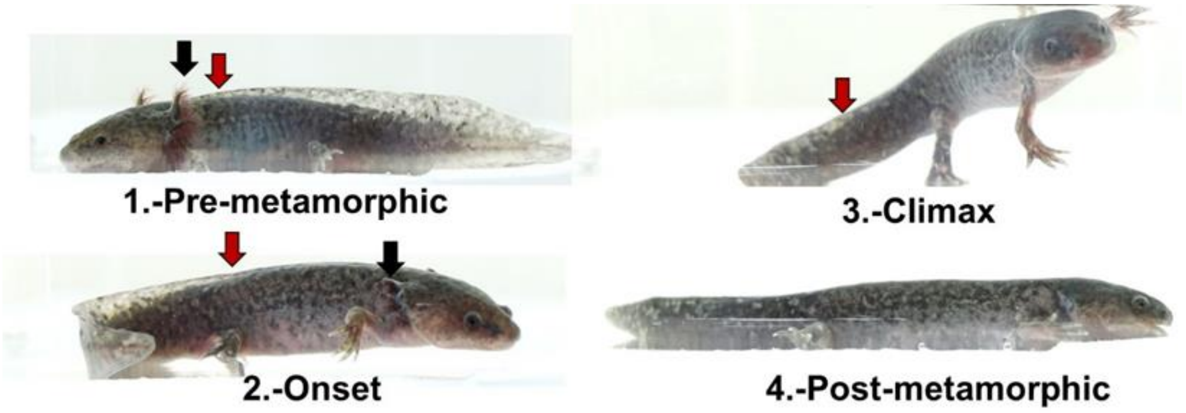

Supplementary Figure 1: Characterization of axolotl metamorphosis stages. We induced axolotl metamorphosis with T3 (500 nM) and followed the morphological changes during the whole metamorphosis process. The stages were characterized as follow: 1, the pre-metamorphic stage, when the experiment initiates and the axolotl has juvenile characteristics such as external gills and large dorsal fin; 2, the onset stage, at ~ day 12 of treatment, where the secondary gills and dorsal fin start to degenerate; 3, climax, at ~ day 20 of treatment, when the external gills were fully absorbed and only an anterior proportion of the dorsal fin remains, and 4, post-metamorphic at ~ day 35 of treatment, when the neotenic phenotype is fully replaced by the post-metamorphic salamander. Arrows denote gills (black) and dorsal fin (red) remodeling during metamorphosis
