# Supplementary material for: Differential effects of 3,5-T2 and T3 on the gill regeneration and metamorphosis of the Ambystoma mexicanum (axolotl)
Source: Front Endocrinol (Lausanne). 2023 Jul 10;14:1208182. doi: 10.3389/fendo.2023.1208182 (PMC10364608; doi:10.3389/fendo.2023.1208182)
Supplement: Supplementary file 6 [file Image_2.pdf]

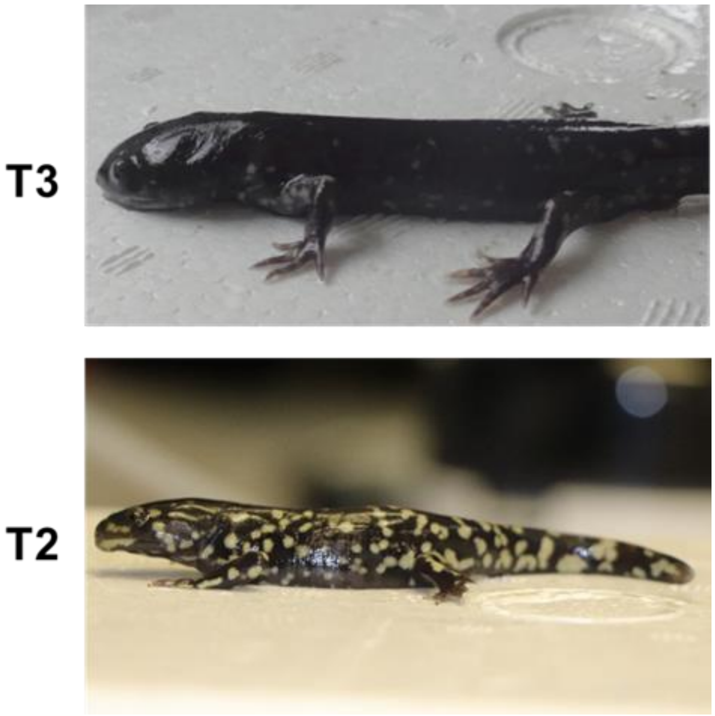


**Supplementary Figure 2.** Phenotype and morphology of the post-metamorphic salamander after T3 and 3,5-T2 treatments. Animals treated with 3,5-T2 showed yellow spotted skin.
