# Supplementary material for: Differential effects of 3,5-T2 and T3 on the gill regeneration and metamorphosis of the Ambystoma mexicanum (axolotl)
Source: Front Endocrinol (Lausanne). 2023 Jul 10;14:1208182. doi: 10.3389/fendo.2023.1208182 (PMC10364608; doi:10.3389/fendo.2023.1208182)
Supplement: Supplementary file 7 [file Image_3.pdf]

A

```
immunogen  DQIILLKGCCMEIMSLRAAVRYDPESDTLTLSGEMAVKREQLKNGGLGVV
hTRα       DQIILLKGCCMEIMSLRAAVRYDPESDTLTLSGEMAVKREQLKNGGLGVV
axTRα      DQIILLKGCCMEIMSLRAAVRYDPDSETLTLSGEMAVKREQLKNGGLGVV
xlTRα      DQIILLKGCCMEIMSLRAAVRYDPDSETLTLSGEMAVKREQLKNGGLGVV
hTRβ       DQIILLKGCCMEIMSLRAAVRYDPESETLTLNGEMAVTRGQLKNGGLGVV
axTRβ      DQIILLKGCCMEIMSLRAAVRYDPESKTLTLNGEMAVTRDQLKNGGLGVV
           ************************:*.****.*****.* **********
```

B

	immunogen	hTRα	axTRα	xlTRα	hTRβ	axTRβ
immunogen	100					
hTRα	100	100				
axTRα	96	96	100			
xlTRα	96	96	100	100		
hTRβ	92	92	92	92	100	
axTRβ	92	92	90	90	96	100

C

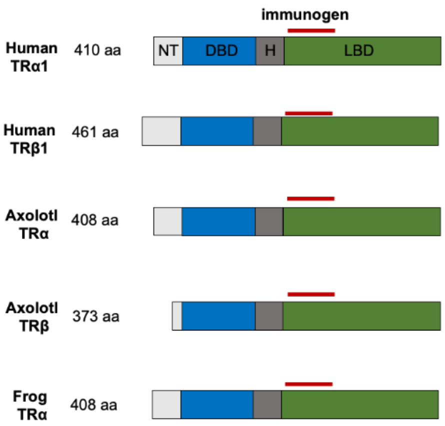

D

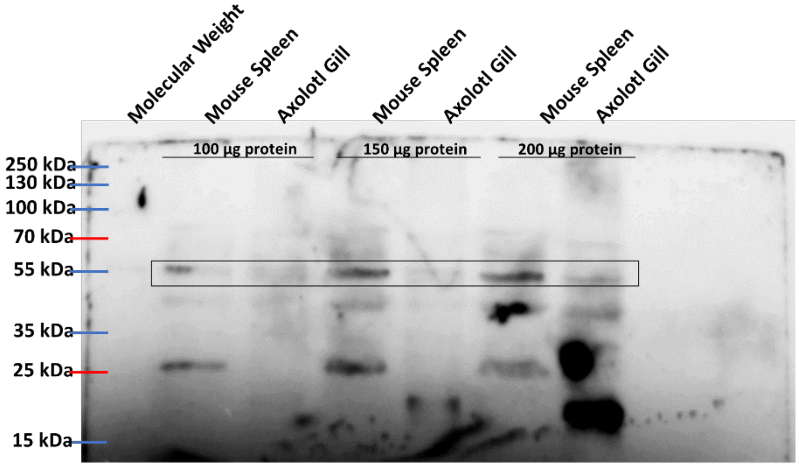

Supplementary Figure 3. The immunogen for the human TR antibody is conserved in axolotl TRα (axTRα) and TRβ (axTRβ). A: Alignment of the amino acid sequences of the immunogen used to generate the Abcam human TR antibody, the human [hTRα (BAH02277.1), hTRβ (P10828.2)], axolotl [axTRα (AAO47436.1), axTRβ (AAO47436.1)] and frog [xlTRα (XP_018089634.1)] thyroid hormone receptor sequences obtained. **B**: Identity matrix for the sequences in **A** showing the percentage of similitude between amino acid sequences. **C**: Topology of the human and axolotl TRs showing the protein site recognized by the antibody (red line). NT: N-terminal Domain; DBD: DNA Binding Domain; H: hinge domain; LBD: Ligand Binding Domain. (*): conserved amino acids; (:) similar biochemical properties; (.): weakly similar biochemical properties. **D:** Immunoblot performed with different concentrations of protein (100; 150 and 200 µg) obtained from mouse spleen as positive control and axolotl gill lysates (using RIPA buffer [Thermoscientific: 89901]) with protease inhibitors (SIGMA: S8830-2TAB) in a 10% acrylamide gel. Abcam primary antibody in a 1:500 dilution and the secondary antibody, HRP-Goat anti Rabbit (Invitrogen: 65-6120) in a 1:3000 dilution. Luminogram was obtained using ECL HRP substrate (Thermoscientific: 32106) and exposed during 25 min in a Amersham Hyperfilm ™ ECL.
